# The Role of Dietary Lipids in Cognitive Health: Implications for Neurodegenerative Disease

**DOI:** 10.3390/biomedicines10123250

**Published:** 2022-12-14

**Authors:** Sakshi Hans, Alexandra Karadimou, John J. E. Mulvihill, Andreas M. Grabrucker, Ioannis Zabetakis

**Affiliations:** 1 Department of Biological Sciences, University of Limerick, V94 T9PX Limerick, Ireland; 2 Bernal Institute, University of Limerick, V94 T9PX Limerick, Ireland; 3 School of Engineering, University of Limerick, V94 T9PX Limerick, Ireland; 4 Health Research Institute, University of Limerick, V94 T9PX Limerick, Ireland

**Keywords:** neurodegenerative disease, dementia, Alzheimer’s disease, functional foods, polar lipids, inflammation, gut-brain axis, gut microbiota

## Abstract

Neurodegenerative diseases are a group of disorders characterised by progressive loss of brain function. The most common of these is Alzheimer’s disease, a form of dementia. Intake of macro- and micro-nutrients impacts brain function, including memory, learning, mood, and behaviour. Lipids, particularly phospholipids and sphingolipids, are crucial structural components of neural tissues and significantly affect cognitive function. The importance of functional foods in preventing cardiovascular disease is well-documented in the current literature. However, the significance of such foods for central nervous system health and neurodegenerative diseases is less recognized. Gut microbiome composition affects cognitive health and function, and dietary lipids are known to influence gut health. Thus, this review will discuss different sources of dietary lipids and their effect on cognitive functioning and their interaction with the gut microbiome in the context of neurodegenerative disease.

## 1. Introduction

The prevalence of neurodegenerative diseases such as Alzheimer’s and Parkinson’s disease is steadily increasing globally. 

The term “neurodegenerative disease” (ND) refers to a group of disorders characterised by progressive loss and degeneration of neurons and neuronal abnormalities. Many NDs can be traced back to the accumulation of proteins such as amyloid-beta (Aβ) and tau in the brain (proteotoxicity), which impair cellular function resulting in cell degeneration and death [1]. These are seen in Alzheimer’s disease (AD), the most common form of dementia [2]. 

ND are age-dependent, with incidence increasing sharply over 60–65 years. Clinical symptoms of dementia include cognitive decline, long-term memory loss, and behavioural abnormalities such as depression, agitation, aggression and sleep disturbances [3]. Among the biological sexes, women are disproportionally affected and show higher rates of AD and other forms of dementia, as well as differing cognitive symptoms [4,5].

Dementia is a major cause of mortality worldwide, with an estimated 1.62 million deaths occurring due to dementia in 2019 [6]. Further, a 2013 investigation reported AD as the sixth leading cause of death in the United States in persons over 65, with the actual figure likely being much higher [4]. The economic and social cost of dementia is also immense, with an estimated annual cost per person of over EUR 32,000 in Europe [7]. Direct costs of dementia include nursing care, drugs, diagnostics, social services, etc. The indirect costs of dementia arise from loss in production due to illness, increased pension, increased morbidity and mortality, impact on families, etc.; it is estimated that these indirect costs of dementia are higher than the direct costs [8].

AD is the most common form of dementia and is estimated to affect as many as 24 million worldwide [2], with the figure estimated to increase steadily with the rapidly ageing population [2]. The prevalence is also much higher with age, with rates sharply increasing over 65 years [2].

Currently, few treatment options exist for ND. For example, the drug Memantine is an NMDA-receptor antagonist used to treat mild-to-moderate AD. However, there is insufficient evidence for its efficacy in treating dementia, particularly in cases of moderate AD [9,10]. In addition, the monoclonal antibody drug Aducanumab has recently been developed to target Alzheimer’s disease by clearing the amyloid plaques in the patient’s brain [11]. However, the clinical efficacy of this treatment is not fully confirmed, and its high cost severely limits its accessibility to patients [12]. Thus, there is an urgent need to explore alternative treatment options and preventive strategies to combat neurodegenerative disease. This narrative review will discuss the pathology of the neurodegenerative disorder Alzheimer’s disease, focusing on the role of neuroinflammation and Aβ disease models based on a critical evaluation of the current literature. The following section discusses the role of the gut microbiome in NDs and the relationship between dietary lipids, gut bacteria, and disease development. We then move on to the role of phospholipids in cognitive health and discuss the current evidence for dietary lipids that promote cognitive health and reduce disease pathology. Finally, we consider the further research required to advance this field of study and fully understand the role of lipids in cognitive health.

## 2. The Pathology of Neurodegenerative Disease—Proteotoxic Insults and the Role of Inflammation and Oxidative Stress

Many NDs are proteinopathies, meaning that the accumulation of specific proteins such as Aβ or tau in the brain tissue is a major hallmark of these disorders. For example, the latter two are typical biomarkers for Alzheimer’s disease (AD) [13]. Amyloid plaques or aggregates are composed of Aβ peptide, which is derived from the abnormal processing of amyloid precursor protein (APP) in the brain [14]. This abnormal processing often arises from mutations in the APP or the enzymes presenilin 1 & 2, which cleave the Aβ peptide from the APP [15].

Parkinson’s disease is the second most common cause of dementia, and it is characterised by the abnormal oligomerisation of alpha-synuclein in neuronal tissue [16]. These aggregates form intraneural deposits known as Lewy bodies and are a characteristic pathologic feature of Parkinson’s disease. These deposits are primarily composed of misfolded aggregates of the protein alpha-synuclein [17].

The presence of amyloid plaques can result in chronic inflammation due to persistent activation of the microglia, a type of immune cells abundantly present in the brain. They play a crucial part in clearing amyloid plaques in the brain, and this phagocytic activity is impaired in AD pathology [18,19]. This leads to the inflammation hypothesis as a pathology of Alzheimer’s disease, which centres on the role of neuroinflammation generated by continuously activated immune cells and the release of neurotoxic substances. While initial activation of immune cells is a beneficial, neuroprotective response, continuous microglial activation can spiral out of control [20] and damage brain tissue by overproduction of pro-inflammatory cytokines such as TNF-alpha, IL-6, and IL-10. Releasing these cytokines and reactive oxygen and nitrogen species (ROS or RNS) causes neuronal damage and death [21]. Newer evidence has also emerged suggesting that soluble and oligomeric forms of Aβ can trigger such activation and subsequent inflammatory response in the brain tissue [22].

An important pathological feature of Parkinson’s disease is the presence of intraneural Lewy bodies. These inclusions have been linked with cytotoxic effects and mitochondrial dysfunction in the brain, although the reports are conflicting [23]. Dementia associated with Lewy bodies has also been linked with neuroinflammation, analogous to amyloid-triggered microglial activation [24,25].

However, the precise mechanisms to explain the neuroinflammatory hypothesis have not been fully elucidated, and information is lacking as to which cells and signalling pathways are involved [26]. 

Closely related to this disease model of AD is the two-hit vascular hypothesis. According to this, initial damage to the cerebrovascular system not only results in neurodegeneration and injury but causes accumulation of Aβ due to a compromised or “leaky” blood-brain-barrier (BBB) [27]. The BBB is a vascular structure that guards against the entry of potentially harmful molecules or pathogens from the bloodstream into the brain and mediates the exchange of certain substances between them. Dysfunction of this barrier can lead to the entry of immune cells and toxins into the central nervous system, which is thus implicated in many pathologies of the CNS [28]. Breakdown of the BBB has been detected in the hippocampi of AD patients and may contribute to cognitive impairment, as found in a 2015 study [29]. An important route by which Aβ is cleared is via transvascular clearance across the BBB. A disruption in this mode of clearance results in neurotoxic accumulation of Aβ in the brain. Transport of Aβ across the BBB is mediated by the LRP1 receptor protein [30], and consequently, expression of LRP1 is altered in AD brains [31]. Peripheral inflammation has been shown to increase Aβ in the hippocampus and cause cognitive impairment in mouse AD models [32]. In another study, inflammation induced by lipopolysaccharide (LPS) in mouse models indirectly affected Aβ clearance and the blood-to-brain influx of Aβ [33]. Deposits of Aβ have been shown to activate the surrounding microglia and cause the production of inflammatory cytokines such as IL6, TNF-α and IL-1β, which play a part in the pathogenesis of AD [34]. Some studies have also shown that metabolites produced by certain microbes can cause Aβ production and thus induce neuroinflammation [35]. Indeed, an imbalance of gut bacteria has been observed in dementia patients [20]. The mechanism of neurodegenerative disease pathology is thus strongly influenced by gut microbiota, which in turn are influenced by the intake of certain dietary lipids, as discussed in the following section.

## 3. Gut Microbiota and the Gut-Brain Axis in Neurodegenerative Disease

**Figure 1:**
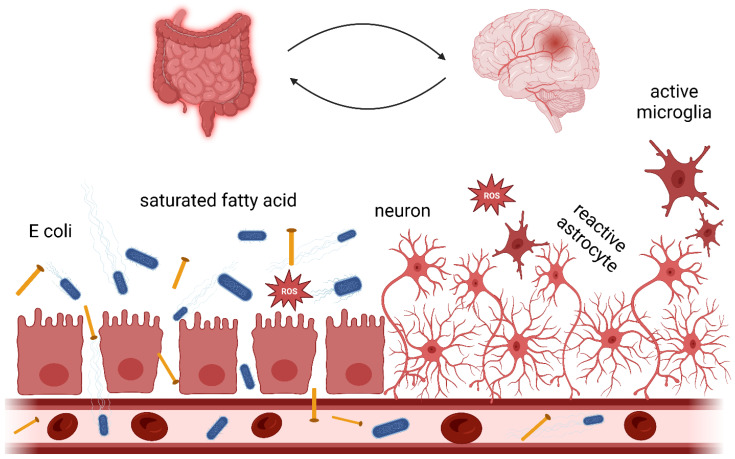
Dietary lipids in the gut-brain axis. Dietary lipids intake can alter gut microbiome balance by increasing the Gram-negative bacteria population [58]. Saturated fatty acid consumption and metabolism affect gut health through reactive oxygen species generation [59]. Chronic gut inflammation can disrupt the intestinal tight junction and consequently lead to a “leaky gut”. Neurotoxins can penetrate the intestinal epithelium and, through the bloodstream, reach the BBB. A compromised BBB facilitates neurotoxins entering the brain and inducing inflammation [53,54]. Active microglia cells and reactive astrocytes can harm neuron cells.

The link between the human microbiome and disease is a rapidly growing field of research. There is increasing evidence for interaction between the gastrointestinal tract (GI) and the central nervous system (CNS). The gut-brain axis can thus be defined as a two-way communication system that functions through pathways such as neural pathways (enteric and sympathetic nervous systems, etc.) and humoral (including cytokine signalling and microbial signalling) [36]. Figure 1 provides a schematic representation of the gut-brain axis. The gut microbiome is a richly diverse community of bacteria that initially colonise the gut following birth and maintain a relatively stable composition throughout life. The human GI tract is estimated to be home to around 200 to 1000 bacterial species [37], with a vast collective genome of almost 200 million genes [38]. Factors such as maternal transmission, host genotype, early-life stress, and diet influence the gut microbiome [39,40,41,42].

Many studies have demonstrated that alterations in the intestinal microbiota composition have important implications for brain function and, consequently, neurological disease. Studies involving germ-free (GF) animal models have found that loss of microbiota impacts cognitive behaviours, anxiety levels and stress response [43,44,45]. The gut microbiota can significantly influence host immune function by modulating the development, proliferation and function of microglia, a type of immune glial cell abundantly found in the brain [46]. For example, microglia from GF-mice display abnormal gene expression and functioning compared to conventionally colonised control mice [47,48]. The gut microbiome is also able to modulate inflammatory signalling pathways such as that of NF-κB ([49,50], TNF-α [51] and interleukin-6 (IL-6) [52]. Imbalance in the gut microbiome has been linked with impaired cognition and abnormal behaviours [53,54]. One proposed mechanism by which this may occur is the passage of neurotoxins through the compromised BBB and/or “leaky gut” [55,56]. Pathogens may also disrupt the BBB and penetrate into the brain by several different mechanisms, including increased production of pro-inflammatory molecules and ROS, receptor-mediated adhesion of bacteria and disruption of endothelial junctions [57].

In one study, germ-free APP transgenic mice (APP PS1) exhibited a significantly altered gut microbiome and APP amyloid pathology compared to the GF wildtype. Faecal microbiota transplantation (FMT) of germ-free mice with bacteria from APP PS1 mice led to increased cerebral Aβ pathology compared to colonization with bacteria from wild-type control mice [58]. 

There is also evidence that molecules produced by gut microbes affect brain functioning and contribute to the development of neurodegenerative disease. For example, gamma-aminobutyric acid (GABA) is an important inhibitory neurotransmitter that regulates anxiety and depression-like behaviours in humans. Certain strains of bacteria, notably Bifidobacterium, can stimulate the production of GABA and consequently influence cognitive functioning [59]. Similarly, Gram-negative bacteria and alterations in their distribution have been linked with AD pathology [60]. 

Imbalance in the gut microbiome is thus significant in the onset and progression of neurodegenerative disease. The composition of the gut microbiome is shaped and altered by dietary intake, and gut microbiota influence lipid metabolism. The dietary fat source also influences gut health [61]. For example, supplementation n-6 and fish oil fatty acids reduced gut inflammation and improved blood-brain-barrier function in mice fed high-fat diets, while supplementation with saturated fats contributed to increased inflammation and insulin resistance [62]. The diversity of beneficial bacteria is also reduced in mice fed a diet enriched with lard or sunflower oils compared to those fed a diet supplemented with fish oil [63,64]. 

Thus, through interaction with host microbiota, dietary lipids may affect the physiological function and brain health. Indeed, a recent investigation found that intake of a multi-strain probiotic formulation significantly improved Aβ pathology and modulated lipid metabolism through inhibited cholesterol biosynthesis and altered omega 6/omega 3 fatty acid ratio [65]. Furthermore, aged microbiota can also significantly alter phospholipid metabolism and composition, with the cortical abundance of the phospholipid subclasses PE and PE found to be changed in mice colonised with aged microbiota. Table 1 lists evidence for the relationship between microbial composition, dietary lipids and cognitive health.

## 4. The Role of Phospholipid Subclasses and Their Biological Functions

**Figure 2:**
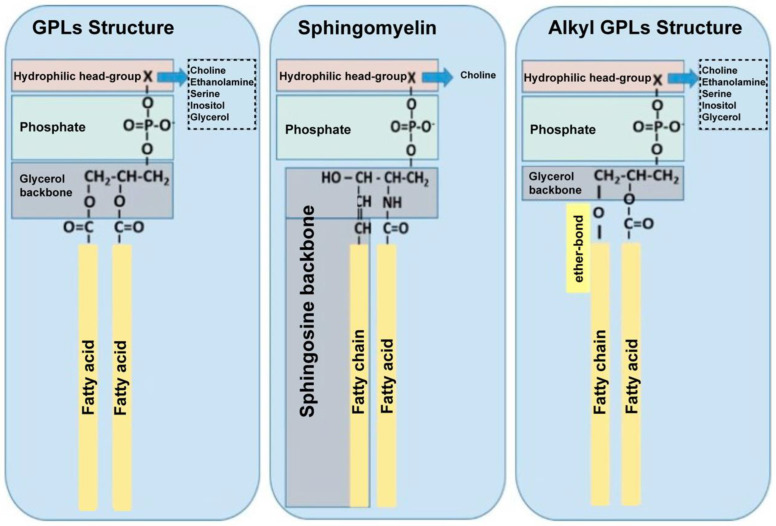
Representation of common phospholipid structures: Glycerophospholipids (GPLs); an example of sphingosine-backbone phospholipid (SPLs), Sphingomyelin; and Alkyl-GPLs, which contain a fatty chain linked with an ether-bond at the *sn*-1 position of the glycerol backbone [71].

The term lipid encompasses various biomolecules such as fats, waxes, glycerides and phospholipids. Lipids can be hydrophobic or amphiphilic by nature, and they have many critical physiological roles in living organisms, including as sources of energy storage, in metabolism and as structural components of biological membranes, including neural membranes [70]. These lipids possess a hydrophilic head and hydrophobic tail and are ubiquitous in cell and organelle membranes as glycerophospholipids (GPLs) (Figure 2). 

### 4.1. Sphingomyelin

Sphingomyelin (SM) lipids are a subclass of phospholipids abundant in biological membranes and essential for the function and development of the nervous system [72]. It is a major structural element of neural membranes and regulates cell growth and differentiation. Infants fed a formula containing milk fat globule membrane (MFGM) enriched with SM were reported with better cognitive development compared to infants fed a standard formula [73,74]. Similarly, preschool children who regularly consumed a milk formula enriched with MFGM polar lipids displayed a lower frequency of febrile episodes, hinting that intake of these PL may improve behavioural regulation [75].

### 4.2. Phosphatidylserine

The acidic phospholipid class known as phosphatidylserine (PS) is enriched in the cerebral cortex and plays a critical role in neural structure and function [76,77]. The distribution of phospholipids across the cell membrane is significant for correct cellular functioning and health, and disruption of the normal asymmetric distribution of PS on the membrane results in the initiation of apoptosis [78,79]. In this regard, levels of PS asymmetry were significantly decreased in the brains of patients with AD and mild cognitive impairment (MCI) [78]. Dietary supplementation with PS and docosahexaenoic acid (DHA) improved oxidative parameters and spatial memory function in rat pups [80], confirming that supplementation is beneficial for the functioning of the developing brain. Another study found that daily intake of soybean-derived PS over 12 weeks in elderly volunteers with impaired memory function resulted in significantly improved outcomes of learning and memory [81]. 

### 4.3. Phosphatidylcholine

Phosphatidylcholine (PC) is an important dietary source of the B-group vitamin choline, a precursor of the neurotransmitter acetylcholine [82]. An ex vivo investigation showed that a higher intake of PC was associated with better cognitive function (as assessed by improved performance on verbal and memory tests) and a lower risk of dementia in middle-aged Finnish men [83]. Another study examined the effect of PC-containing diets (including on the cognitive function of male BALB/c mice, with dementia-like characteristics induced by injection with scopolamine [84]. It was found that PC intake could effectively attenuate brain damage caused by treatment with scopolamine, as assessed by cognitive tests such Morris water maze task [84].

Region-specific differences in the spatial localisation of lipid molecules, including phosphatidylcholine, have been detected in AD mouse models compared to wild-type control mice [85]. This indicates that alterations in the spatial localisation of lipids in the brain play an important role in the pathology of neurodegenerative disease and lipid levels. Further, metabolomics profiling identified significant changes in phosphatidylcholines in the *APOE* ɛ4 genotype in AD patients [86].

Ether lipids and phospholipids are of major physiological significance, forming a component of the biological membrane and being involved in signalling pathways. Examples of this class of lipids include platelet-activating factor (PAF), alkylglycerols and plasmalogens. The levels of ether lipids have been linked with neurodegenerative disease. Analysis of brain lipid content in human patients [87] and AD mouse models have found that levels of certain plasmalogen species are significantly altered [88]. Aside from AD, Parkinson’s disease has also been associated with altered lipid levels. A clinical investigation of Parkinson’s disease found that oral administration of purified ether phospholipids improved clinical symptoms [89].

Consumption of fish-derived omega-3 polyunsaturated fatty acids (PUFAs) has been linked with a lower incidence of subclinical brain abnormalities in older adults [90]. For example, the autosomal dominant neurodegenerative disorder Huntington’s disease is characterised by severe cognitive decline and dementia. In addition, a study has shown that the administration of deuterium polyunsaturated fatty acids (D-PUFA) significantly improved cognitive decline through antioxidant activity and reduction of lipid peroxidation [91].

A recent study using rat models showed that milk fat globule membrane supplementation during pregnancy promotes neurodevelopment in offspring by modulating the gut microbiome and downregulating levels of pro-inflammatory cytokines and lipopolysaccharide (LPS) in the circulation [92]. Similarly, oral supplementation of phosphatidylserine (PS) improved learning and memory function in young rats and upregulated the production of the neurotrophic factors BDNF and IGF-1 in the hippocampus [93]. Moreover, a 2022 study demonstrated that the omega-6 PUFA linoleic acid is highly neuroprotective and reduces inflammation in in vitro models of Parkinson’s disease [94]. 

Table 2 summarises these and other in vitro and in vivo investigations where lipids improved cognitive health and function.

### 4.4. Current Knowledge: Types of Fatty Acids of Lipids and Their Role in Neuroprotection

Lipids are involved in several critical signalling pathways, acting as signalling activators, mediators and enzyme substrates. Membrane lipids are crucial components of signal transduction pathways, which are often highly interconnected and interconvertible [106]. The phosphatases and lipid kinase molecules are some of the most important signalling components. Phosphoinositide 3-kinase (PI3K) is a prominent example of a lipid phosphorylation pathway since mutations in this pathway have been associated with cancer [107]. Lipid molecules can also act as mediators and bind G-protein coupled receptors (GCPRs) on the cell surface. These include phosphoglycerides, leukotrienes, lysophospholipids, and the phospholipid platelet activating factor (PAF). PAF binds the PAF receptor and triggers downstream activation, thereby regulating processes such as the immune response, cell proliferation and apoptosis, making it an important mediator of these downstream signalling events [108].

The sterol class of lipids, particularly cholesterol, are essential components of cellular membranes. Cholesterol is a major structural component of lipid rafts, and thus its metabolism strongly influences membrane fluidity [109] and several membrane signalling pathways [110,111]. Cholesterol has recently been shown to interact with scaffold proteins such as NHERF1 and thus regulate cellular signalling and trafficking pathways [112].

Saturated fatty acids (SFA), such as palmitic acid and stearic acid, have significant roles in cognitive function. Several studies have shown that palmitic acid can induce neuroinflammation and microglial activation, thus promoting neurodegeneration [113,114,115]. On the other hand, stearic acid seems neuroprotective [116]. For example, a recent study reported that hydroxy stearic acid (5-PAHSA) is neuroprotective in vitro and in vivo [117]. Thus, these SFA differentially impact neurological health and function.

Monounsaturated fatty acids (MUFA) also have a beneficial impact on brain health. For example, oleic acid is an omega-6 MUFA enriched in oils such as olive oil. Intake of this fatty acid has been shown to protect against oxidative stress in vivo [118] and reduce Aβ secretion in APP-transgenic mice [119]. Similarly, a 2018 study reported that deuterium-reinforced linoleic acid in the diet of Huntington’s disease model mice caused reduced lipid peroxidation and improved performance in cognitive tests [91]. Another study in rat models of multiple sclerosis reported improvement in oxidative stress parameters in the brain of rats fed conjugated curcumin-linoleic acid, as well as improved memory scores [120].

Dietary lipids are hydrolysed by pancreatic lipases in the lumen of the intestine and taken up through transporter proteins located in enterocytes, the absorptive cells of the gut. Before this, however, the lipids are emulsified by bile salts to facilitate their hydrolysis into products such as free fatty acids, glycerols and monoglycerides. Following uptake by enterocytes, these hydrolysed products are transported to the endoplasmic reticulum (ER), where they are resynthesized into lipids by processes such as esterification. These lipids are subsequently either stored as lipid droplets in the cytosol or secreted out of the ER in the form of lipoprotein particles known as chylomicrons [121].

Lipids are abundantly present in the nervous system, with hundreds of lipid species present in the neural tissues. Polar lipids, notably phospholipids and sphingolipids, form major structural components of cellular membranes, including neural tissues. Neural membranes are rich in long-chain saturated fatty acids (LC-SFA) palmitic and stearic acids. Many studies have confirmed the importance of polar lipids in early neurodevelopment and cognitive function. In addition to acting as cell membrane components, polar lipids have roles in vesicular trafficking, signalling transduction and synaptic plasticity [122,123]. Sphingolipids are particularly enriched in the brain, and studies have shown that the profile of sphingomyelin in the brain shifts with factors such as ageing and dietary intake of lipids [124,125,126].

It has been shown that PUFAs are relatively less abundant in brain tissue than in other organs, with MUFAs significantly enriched in the brain [127]. Saturated fatty acids are also abundant in the brain and play structural and metabolic roles in neural tissues; as lipid raft components in plasma membranes and cell signalling molecules in pathways such as NF-κB [128].

The relationship between AD pathology and a high-fat diet (HFD) or obesity is not fully clear, with some studies suggesting that obesity promotes Aβ pathology [129,130]. At the same time, other evidence points towards an HFD as protecting the BBB and promoting cognitive function [131,132,133,134]. 

It is established that systemic inflammation is linked to ageing and worsened cognitive function. The inflammatory potential of diet is thus able to predict the risk of incident dementia [135] and highlights the importance of preventive dietary interventions in reducing the incidence of dementia and other chronic diseases. Indeed, numerous investigations have shown that dietary intake of foods containing beneficial fats with antioxidant and anti-inflammatory properties has a neuroprotective effect and promotes cognitive function (Table 2). It appears that dietary lipid source is a critically important factor in health outcomes, with n-6 and n-3 long-chain polyunsaturated fatty acids (LC-PUFA) having important neuroprotective properties [136,137], while saturated fats negatively impact brain health and contribute to neuroinflammation [138]. An epidemiological study reported that intake of n-3 PUFAs, especially α-linolenic acid, was inversely associated with ND-related mortality [139]. Notbaly, studies have demonstrated the clinical significance of phospholipid subclasses phosphatidylcholine and phosphatidylethanolamine levels in the brain. Ageing has been linked with decreased levels these phospholipids in brain regions of elderly subjects [140]. Moreover, it appears that phospholipid supplementation positively affects memory and stress performance, as found in human studies [141,142].

Supplementation with the omega-3 PUFA docosahexaenoic acid (DHA) and eicosapentaenoic acid (EPA) is beneficial for mood disorders and neurodegenerative disease. EPA and DHA are known to promote neurite outgrowth and affect synaptic plasticity in rat hippocampal neurons [143,144]. Dietary supplementation with EPA and DHA has been shown to improve age-related inflammatory signalling and cognitive function [80,145,146]. Many studies have demonstrated the role of omega-6 PUFA, such as linoleic acid and arachidonic acid, in neurodegeneration and neuroinflammation. It appears that the intake of linoleic acid, a PUFA abundant in corn and soybean oils, is neuroprotective and anti-inflammatory in models of Parkinson’s disease (PD) [94,147]. Arachidonic acid is an omega-6 PUFA mainly found in animal sources, such as meats, poultry, eggs and seafood, and plays important roles in membrane fluidity, neurodevelopment and neuronal signal transduction [148]. Similar to previously discussed lipids, arachidonic acid has neuroprotective properties. For example, it inhibits cytotoxicity in vitro in cell models of PD [147]. Another study reported that dietary supplementation with arachidonic acid added to DHA improves impairment in social interactions in children with autism spectrum disorders [149].

The lipid class of sterols is abundantly present in the brain, and many sterols possess neuroprotective properties. For example, cholesterol metabolism and homeostasis are closely linked with brain health and function. Cholesterol levels in the brain affect crucial processes such as neurodevelopment and neuronal signalling. Evidence also suggests that dysregulated cholesterol homeostasis contributes to neurodegenerative disease pathology [150,151]. The role of phytosterols such as stigmasterol, campesterol and β-sitosterol in reducing ND pathology has been highlighted in recent investigations. This group of sterols are naturally present in plants, which possess neuroprotective and cholesterol-lowering properties and can cross the blood–brain barrier, thereby contributing to cognitive health. A 2013 study showed that consuming stigmasterol-enriched diets in mice leads to reduced Aβ production, indicating that intake of this phytosterol protects against AD pathology [152]. Dietary intake of marine sterols such as fucosterol, a compound derived from brown algae, is noted for its antioxidant [153] and anti-inflammatory properties [154]. Fucosterol has also been shown to decrease Aβ oligomer aggregation and thus reduce Aβ pathology in AD [155]. Another sterol compound derived from seaweed with similar neuroprotective properties is saringosterol, derived from *Sargassum fusiforme.* In AD model mice, dietary supplementation with 24(S) saringosterol prevented memory decline and reduced Aβ deposition [156]. Thus, phytosterols also represent an important source of anti-inflammatory lipids that can be adopted as part of a dietary regimen to combat neuroinflammation.

Oxysterols are various oxidation products of sterols, a prominent example of which is 7-ketosterol (7-KC). 7-KC is a toxic compound that leads to deleterious processes, such as the generation of reactive oxygen species (ROS) and cellular death and damage [157], and has been found in arterial plaques and other disease tissues. There is evidence that 7-KC contributes to AD pathology by causing microglial dysfunction and impaired clearance of amyloid plaques [158,159]. 7β-hydroxycholesterol (7β-OHC) is another cholesterol oxidation product like 7-KC, and similarly to 7-KC, is also implicated in age-related disease and inflammatory processes. However, studies have shown that it is similar to counter the harmful effects of these oxysterols, including cytotoxicity and mitochondrial stress, by consumption of nutrients such as tocopherols [158,160,161]. Alpha and gamma-tocopherols can cross the blood–brain barrier and counter the neurotoxic effect of oxysterols [162,163,164]. The impact of sterols on brain health is thus quite significant, with beneficial phytosterols conferring neuroprotection, although oxidation products like 7-KC are deleterious and neurotoxic.

### 4.5. Lipids and Human Disease

The current knowledge of dietary lipids and ND suggests a positive impact on disease pathology. A study with AD mouse models found that maternal supplementation with DHA-enriched fish oil improved cognitive function and prevented neuronal dysfunction in the cortex [165]. Similarly, another study found that DHA supplementation reduces AD pathology and Aβ oligomer aggregation [96]. An investigation of Parkinson’s rat models found that pre-treatment with DHA protected against dopaminergic neuronal death [166]. A similar study with PD mice showed that fish oil supplementation decreased lipid peroxidation and improved dopaminergic neuronal turnover [95]. 

Oils containing tocopherols, such as alpha-tocopherol, are enriched in the Mediterranean diet. These are rich in oleic acid and linoleic acid and have significant antioxidant properties. There is ample evidence for many tocopherols, most notably olive oil, in preventing Alzheimer’s disease. One population study [167] found that the consumption of olive oil was linked with a lower risk of death and protection from cognitive decline and stroke. This may be attributed to the highly bioactive phenolic compounds present in olive oil, such as oleuropein aglycone and oleocanthal, which can reduce Aβ pathology and decrease neuroinflammation and oxidative stress [168,169,170]. Indeed, the intake of extra virgin olive oil has been proven to improve cognitive performance in subjects with mild cognitive impairment [171]. 

Argan oil is another type of highly bioactive vegetable oil abundant in alpha-tocopherols. Studies in rat models have demonstrated the potential of argan oil to protect against certain neuropsychiatric disorders due to its ability to inhibit oxidative stress, improve mitochondrial function and modulate inflammation [172]. One study using 158 N murine oligodendrocytes as a neurodegeneration model reported that argan oil treatment reduced 7-KC-induced cytotoxicity [173]. Milk thistle oil is another primary source of alpha tocopherols, and it is similarly reported to ameliorate the oxidative stress effects of oxysterols such as 7β-hydroxycholesterol [174], which are increased in patients with ageing-related diseases [175].

A number of important studies highlighting the effect of lipid supplementation or treatment in in vitro and ex vivo studies are detailed in Table 2**.**

There is also some evidence for supplementation with dietary lipids to improve the pathology of Huntington’s disease (HD), a rare autosomal dominant disorder characterised by progressive loss of nervous system function. Indeed, dysregulation in lipid metabolism and increased insulin resistance have been linked to the disease [151,176]. In a mouse model of HD, omega-3 fatty acids such as EPA have improved motor function (but not neurodegeneration) [177]. However, the evidence so far is insufficient to draw a conclusion about the role of dietary lipids in HD, and studies with greater sample sizes and more robust designs are required to fill the research gap.

## 5. Conclusions and Future Perspectives

Fats have been popularly regarded as mainly detrimental to health and contributing to chronic disease. However, much evidence from dietary studies shows that many functional foods are rich sources of beneficial lipids that support cognitive function. For example, beneficial lipids such as those abundant in the Mediterranean diet (such as olive oil and fish oils) can boost cognitive performance in AD models. The impact of dietary fat on health and disease depends on factors such as lipid source and balance of omega-6 and omega-3 fatty acids. These functional lipids, most prominently EPA and DHA, are present in different sources such as dairy, fish and vegetable fats. Therefore, supplementation with such functional foods should be considered an essential preventive measure to reduce the incidence of cognitive impairment and improve mental performance. Furthermore, a balanced intake of unsaturated lipids in the diet positively impacts the functioning of the brain, gut microbiome, and cardiovascular system.

Further investigations are required, however, to clarify how lipid metabolism is affected through gut-brain signalling. Future studies would also aim to understand the specific mechanisms by which dietary lipids may prevent or improve ND pathology, for example, by reducing neuroinflammation of the generation of reactive oxygen species (ROS) in the brain. Following on from here, efficacious and highly targeted lipid formulations must be developed through rigorous clinical testing and development to reduce cognitive impairment. Finally, further understanding of the transport mechanisms of LC-FAs across the blood-brain barrier is required to facilitate interventions to prevent the incidence of neurodegenerative diseases.

## Figures and Tables

**Table 1:** Study evidence for lipids and gut-brain signalling: Microbe-dietary lipid interaction.

Study Aim	Lipid Type	Microbes	Study Type	Study Result	Study Reference
Understand the effect of fish oil (FO) intervention on gut dysbiosis and neuropsychiatric behaviours in rat models of human geriatric depression	FO containing n-4 PUFA	Bacteroidetes, Prevotellaceae, Marinifilaceae, and *Bacteroides uniformis*	In vivo study with Sprague Dawley rats	FO intake in rats improved emotional symptoms of depression and a reduced load of certain bacterial taxa	[66]
Assess the effect of long-term supplementation of n-3 PUFA on gut dysbiosis due to early-life stress	n-3 PUFA mixture (80% DHA and 20% EPA)	Bacteroidetes and Firmicutes	In vivo study with rat models	The abundance of the phyla Bacteroidetes and Firmicutes was altered in maternally separated (MS) rats compared to non-separated rats, and this dysbiosis is rescued upon long-term EPA/DHA administration	[67]
Study of gut microbiota composition in children with neurodevelopmental disease (NDD) and evaluation of Short-chain fatty acids (SCFA) levels	Short-chain fatty acids	Potentially harmful bacteria, such as *Desulfotomaculum guttoideum* and *Intestinibacter bartlettii,* and benign bacteria, including *Enterococcus* and *Lactobacillus*	Ex vivo, placebo-controlled study on preadolescent children diagnosed with NDDs.	Microbial diversity was decreased in NDD patients compared to control. Increased prevalence of harmful bacteria, including *Desulfotomaculum guttoideumIntestinibacter bartlettii and Romboutsia ilealis*, and lower prevalence of commensal bacteria in the gut	[68]
Investigate the effect of long-term adherence to a Mediterranean diet (MD) on gut microbiome and frailty in older adults	Mediterranean diet (vegetables, legumes, fruits, nuts, olive oil and fish and low consumption of red meat and dairy products and saturated fat	*Faecalibacterium prausnitzii*, *Eubacterium* and *Roseburia*, *Ruminococcus torques*, *Collinsella aerofaciens*, *Coprococcus comes*, *Dorea formicigenerans*, and *Clostridium ramosum.*	Randomised single-blind controlled dietary intervention study	Gut microbial composition was altered following intake of MD. A lower frequency of bacterial taxa associated with markers of ageing and inflammation was observed, and taxa associated with improved cognitive function were enriched in the gut	[69]

**Abbreviations:** DHA, Docosahexaenoic acid; EPA, Eicosapentaenoic acid; FO, fish oil; MS, maternally separated; MD, Mediterranean diet; NDD, neurodevelopmental disorder; PUFA, polyunsaturated fatty acids; SCFA, short-chain fatty acids.

**Table 2:** Dietary supplementation with functional lipids improves cognitive function (human trials and animal studies).

Study Aim	Functional Lipid Source	Study Type	Study Result	Study Reference
Assess the neuroprotective effects of linoleic acid in the SH-SY5Y PD cell line and a PD mouse model	Linoleic acid	In vitro cell culture model and in vivo mouse study	Administration of LA confers protection from neuroinflammation and neurodegeneration in vivo. LA also shows anti-inflammatory and antioxidant properties in vitro.	[94]
Determine whether docosahexaenoic acid (DHA) and phosphatidylserine (PS) supplementation can improve the cognitive function of the developing brain and reduce oxidative stress	Docosahexaenoic acid (DHA) and phosphatidylserine (PS)	In vitro study with C6 glioma cells and in vivo with rat pups	Supplementation with DHA and PS significantly improved antioxidant activity in vitro and in vivo and also improved learning and memory parameters in rat models	[80]
Determine the effect of omega-3 supplementation on 6-hydroxydopamine Parkinson’s disease models	Fish oil containing omega-3 fatty acids	In vivo study with rats	Omega-3 supplementation resulted in increased dopaminergic neuron turnover and improved performance in behavioural tests	[95]
Study the effect of dietary DHA supplementation on APP/PS1 transgenic Alzheimer’s disease rat models	DHA-supplemented diet	In vivo study with rat models	Rats fed supplements exhibited a lower density of amyloid plaques, improved behavioural performance, and reduced Aβ aggregation.	[96]
Study the effect of fish oil (FO)and blueberry (BB) supplementation in older adults with self-reported cognitive decline	FO enriched with DHA + EPA and BB	Dietary trial in older adults	After 24 weeks of supplementation with FO and BB, subjects experienced impairment in memory and daily functioning.	[97]
Investigate the impact of dietary intake of soy lecithin supplement on AD symptoms in elderly patients	Soy lecithin supplement containing PS (300 mg/day) + PA (240 mg/day)	A double-blinded placebo-controlled study with elderly patients with AD	Dietary supplement positively impacts memory, cognitive function and mood in AD patients.	[98]
Evaluate the effect of different dietary regimens with EPA, DHA and combinations of these on dementia symptoms	Dietary supplement of DHA/EPA and combination of EPA + DHA	A randomized, double-blind, placebo-controlled trial in elderly patients with mild cognitive impairment (MCI) or AD	EPA intake improved scores in spoken language tests, although no statistical improvement in mood, cognitive function and other parameters was observed. EPA significantly reduced levels of CCL4, an inflammatory biomarker for cognitive decline	[99]
Study the effect of combined supplementation with three different nutrients proven to benefit cognitive health (fish oil, carotenoids and vitamin E)	1 g fish oil (containing 430 mg docosahexaenoic acid, 90 mg eicosapentaenoic acid), 22 mg carotenoids and 15 mg vitamin E	Randomised, placebo-controlled human trial in healthy older adults	After a 24-month supplementation period, subjects showed significant improvement in working memory performance compared to the placebo	[100]
Assess the effect of bovine milk-fat globule membrane (bMFGM) intake on infant neurodevelopment	Bovine milk-fat globule membrane	Randomised double-blinded placebo-controlled human trial	Infants who received formula supplemented with bovine MGFM exhibited improved neurodevelopmental profiles, including higher language and motor scores, compared to infants fed a skim milk-based control	[74]
Assess the impact of dietary intake of Greek high phenolic early harvest extra virgin olive oil on cognitive performance in subjects having mild cognitive impairment	Greek high phenolic early harvest extra virgin olive oil	Randomized clinical trial	Dietary intervention with high phenolic early harvest extra virgin oil was associated with improved performance in neuropsychological battery test compared to volunteers fed only a Mediterranean diet, independent of APOE ɛ4.	
Assess the effect of soybean-derived PS (SB-PS) intake on cognitive performance in elderly	Soybean-derived phosphatidylserine (SB-PS) (dosage: 300 mg/day)	Single-centre, open-label, placebo-controlled human study	Supplementation with SB-PS significantly improved cognitive parameters, including memory, learning and executive function in volunteers compared to control	[81]
Determine the effect of intake of coconut oil-enriched Mediterranean diet on cognitive function in AD patients	Coconut oil-enriched Mediterranean diet (MD)	A prospective qualitative study in human patients	Consumption of coconut oil-enriched MD improved cognitive function in AD patients compared to the control group	[101]
Evaluate the effect of intake of a Mediterranean diet enriched with olive oil and nuts on cognitive performance	Mediterranean diet supplemented with olive oil and mixed nuts	Randomised controlled trial	Improved scores in tests of cognitive function were observed in cohorts taking either MD plus olive oil or MD plus nuts versus the control group	[102]
Investigate the relationship between dietary intake of total fat and cognitive impairment in older Chinese adults	Total fat intake classified into plant-based fat and animal fat	Ex vivo population-based cohort study	Higher dietary intake of total plant-based fat was linked with decreased risk of cognitive impairment in middle-aged Chinese adults	[103]
Determine the association between long-chain fatty acid (particularly EPA and DHA) intake and cognitive impairment as a 13-year dietary intake study	Long-chain n-3 FA and fish	Dietary assessment study	Low cognitive test scores were negatively associated with a high long-chain FA, DHA and EPA intake. High fish consumption was significantly linked with a lower frequency of cognitive complaints after adjustment for depressive symptoms	[104]
Evaluate the efficacy of fish oil omega-3 DHA supplementation on mental performance in schoolchildren using a series of cognitive tests	Fish oil DHA capsule supplement (low dose 260 mg DHA and high dose 520 mg DHA)	Randomised double-blinded placebo-controlled clinical trial	Regular intake of FO resulted in higher mental ability, including better attention scores and cognitive processing, compared to baseline	[105]

**Abbreviations**: AD, Alzheimer’s disease; BB, blueberry; bMFGM, bovine milk fat globule membrane; DHA, Docosahexaenoic acid; EPA, Eicosapentaenoic acid; FO, fish oil; LA, Linoleic acid; MD, Mediterranean diet; MCI, mild cognitive impairment; PS, phosphatidylserine.
